# Formation of configurable uniform CdSeTe thin films by close-space sublimation deposition of multiple alternating CdSe and CdTe layers

**DOI:** 10.1080/14686996.2026.2633815

**Published:** 2026-02-26

**Authors:** Pascal Jundt, Olaf Zywitzki, Thomas Modes, Sagar Baitule, Robert Arndt, Bettina Späth, Bastian Siepchen

**Affiliations:** a Research and Development, CTF Solar GmbH, Dresden, Germany; b Materials Science/Analytics, Fraunhofer Institute for Electron Beam and Plasma Technology FEP, Dresden, Germany

**Keywords:** Cadmium telluride, cadmium selenium telluride, close-space sublimation, recrystallization, intermixing, porosity

## Abstract

Incorporation of selenium within cadmium telluride to form the CdSe_x_Te_1-x_ alloy has enabled higher device efficiencies in photovoltaic applications through improved passivation and current collection. Recent investigations of this alloy have simultaneously indicated significant potential for further performance gains as well as potentially serious detrimental characteristics. Exploring how best to utilize this important material requires consistent, configurable film deposition. Absorber layers are often deposited by close-space sublimation, which can produce high-quality films at large scale. It has previously been demonstrated that close-space sublimation of CdSeTe material directly is inconvenient and inflexible; therefore, an alternative method with greater consistency and control over film composition is sought. In this work, CdSeTe films were formed by close-space sublimation of alternating CdSe and CdTe layers followed by a high-temperature annealing in the presence of CdCl_2_. Under the optimal deposition conditions, this technique was shown to produce high-quality films of CdSeTe with homogeneous elemental distribution, uniform grain size, and low roughness. However, this method also exhibited a significant tendency to form numerous voids which largely persist after CdCl_2_ annealing. The source of this porosity was investigated and determined to primarily be resublimation of CdTe during the higher-temperature deposition of the CdSe layers. The process window to prevent voiding was observed to be rather small; while this would be a significant detriment in a production setting, it is less important in the targeted application for this approach, which is fundamental material and device investigations.

## IMPACT STATEMENT

This manuscript describes a multilayer-based method for depositing thin films of the photovoltaic alloy CdSeTe with precise control of elemental composition, which provides research-supporting flexibility not afforded by traditional techniques.

## GRAPHICAL ABSTRACT

**Figure:**
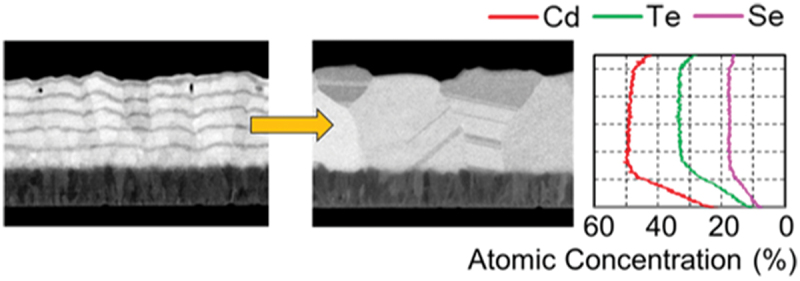

## Introduction

Cadmium telluride has been the leading thin-film photovoltaic technology in terms of installed capacity for almost two decades [1]. During this time, several advancements pertaining to doping, material quality, and light collection have increased conversion efficiencies significantly in both small-area research devices and production modules [2,3]. One such development is the incorporation of the ternary CdSeTe alloy within the absorption region of the device stack, improving passivation and increasing current collection [4–6]. Current state-of-the-art CdTe technology utilizes a CdSeTe/CdTe bilayer absorber, with a thicker CdTe layer [7]. Absorber deposition is possible with a variety of methods. Close-space sublimation (CSS) [8–10] is particularly common as it can produce good-quality films with high throughput and is readily scalable and adaptable as an in-line process.

CSS deposition of the CdSeTe layer directly from the CdSeTe source material is associated with a host of complications arising from the different vapor pressures of the two constituent binary species at sublimation temperatures. CdTe sublimates faster than CdSe at a given temperature, leading to gradual changes in source material composition over time. Ensuing consequences include continually evolving deposited layer composition, structure, and deposition rate, even if all process parameters are kept constant [11,12]. Small compositional inhomogeneities in the source are amplified over time. Complications are more severe with higher CdSe fractions. Such inconsistency is inconvenient in a research context and thoroughly unacceptable in a production setting. Deposition of CdSeTe from a mixture of CdTe and CdSe powders by CSS [13] and thermal evaporation [14] has also been investigated. This affords a higher degree of composition adjustment than direct vaporization of CdSeTe, although switching to a different selenium concentration still requires a complete exchange of source material, and the unstable stoichiometry of source material over time is not resolved.

A high-temperature anneal with CdCl_2_ has long been a crucial processing step in CdTe photovoltaics to recrystallize and passivate grains deposited by virtually every deposition method [15–19]. This anneal is also very effective at diffusing selenium and tellurium [4,20–22]. Therefore, graded CdSeTe layers in state-of-the-art devices are sometimes formed by CdCl_2_ annealing of CdSe/CdTe bilayers [12,23,24]. Some degree of control over this grading can be exerted through the thickness of the CdSe layer and the CdCl_2_ annealing conditions. Typically, the CdSe thickness is much less than CdTe – on the order of tens to no more than a few hundred nanometers – to ensure complete decomposition of CdSe, as even a very thin remnant CdSe layer reduces current through parasitic absorption and buried junction formation [21,25–27]. This ultimately limits the flexibility of this architecture, constraining CdSeTe layer thickness and guaranteeing a graded composition of selenium.

The ease of elemental diffusion suggests another approach for CdSeTe film preparation. This manuscript investigates the formation of uniform CdSeTe films through deposition of alternating CdSe and CdTe layers and subsequent recrystallization during a CdCl_2_ annealing treatment. This novel technique has several potential benefits. As the binary alloys are sublimated separately, the inconsistency associated with CdSeTe sublimation is eliminated. The fraction of selenium in the deposited CdSe_x_Te_1-x_ alloy is easily controllable by the thicknesses of the CdSe and CdTe layers and is not fixed by the selenium fraction of the source material itself. Selenium fraction can therefore also be easily adjusted on a sample-by-sample basis without needing to exchange source material, a time-consuming process which requires breaking vacuum in the deposition chamber. As will be demonstrated, high-quality films with favorable grain structure and high elemental uniformity can be created with this method.

CdSeTe is relatively poorly understood, and its properties are a current major area of study. While incorporation in a bilayer absorber structure yields consistent cell performance improvements, CdSeTe has additional signifiers of substantial further potential improvements in device performance when used as the sole absorber material. Very high lifetimes and photoluminescence quantum yields have been reported [28,29], indicating a material which can support voltages >150 mV higher than currently typically attained in cells. At the same time, however, the more recent discovery of shallow radiative defects attributable to selenium alloying and doping with group V elements such as arsenic [14,30,31] signifies loss mechanisms which may or may not fatally harm the potential use of this material in a uniform composition absorber. The amount of selenium appears to have a strong effect on the intensity of all of these behaviors. Therefore, to better understand selenium’s influence and to determine how to best utilize this ternary material, it is important to have a reproducible method for deposition of uniform films with precise control over stoichiometry, as is afforded by the technique described here. Previous investigations of these effects have generally been limited to a small number of discrete CdSe fractions. The multilayer approach would facilitate examination of a larger number of discrete compositions with fine variation between them, with additional capabilities of high throughput for statistical analysis and potential future applications to large-area devices. In a more conventional application, this approach can also be used to deposit films for the CdSeTe/CdTe bilayer structure, but without the customary restrictions of thickness and grading.

## Methods

In this investigation, layer stacks comprised of CdSe and CdTe were deposited by close-space sublimation (CSS) in several configurations. Commercially available glass substrates with transparent conductive oxide (TCO) films were oscillated proximal to CdTe and CdSe sources to create film stacks with multiple alternating CdTe and CdSe layers. The CdTe source temperature was generally in the range of ~630–650°C while the CdSe source was in the range of ~690–720°C. CdSe’s higher standard enthalpy of sublimation [32] necessitates consistently higher source temperatures than for CdTe, which complicates the multilayer stack approach, as will be described. Film thicknesses are controlled in large part by source temperature, and also by substrate transport speed. X-ray fluorescence (XRF) spectroscopy is used as a fast ex-situ monitoring technique during deposition to determine the thicknesses of each material within the stack, so the CSS parameters can be adjusted accordingly to achieve the desired composition. Deposition atmosphere was 5 Pa nitrogen for all samples.

All layer stacks were investigated in an as-deposited condition. A few samples were additionally investigated after various CdCl_2_ annealing treatments. In this procedure, a solution of CdCl_2_ in methanol was applied to the back surface of the absorber and left to dry, followed by a high temperature (>420°C) anneal in a furnace, in air. Annealing times and temperatures vary and are described where relevant.

Sample cross-sections were prepared by argon ion polishing using a broad ion beam preparation technique (Jeol, Japan SM-09010). Absorber cross-sections were imaged with field emission scanning electron microscopy (FE-SEM) (Hitachi, Japan SU8000) using material and crystal orientation contrast of backscattered electrons. Elemental profiles through the films were obtained with glow-discharge optical emission spectroscopy (GD-OES) (Horiba, Japan GD Profiler 2), in which the sample is gradually sputtered as an electrode in a glow discharge, and elemental composition of the removed material is simultaneously determined from optical emission analysis. X-ray diffraction (XRD) analysis (Bruker, United States D8 Discover) was used to investigate crystalline phases and lattice parameters. X-ray fluorescence (XRF) (Fischer, Germany Fischerscope X-Ray DDV-SDD) was used to measure element quantity.

## Results

### Epitaxial growth and presence of voids

**Figure 1.**
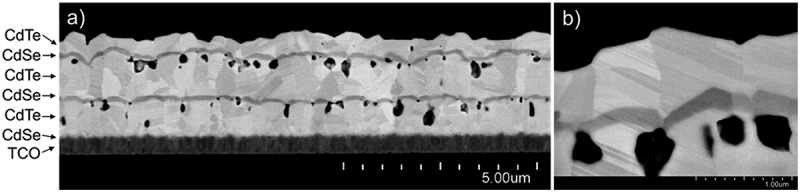
a) Cross-section of characteristic as-deposited multilayer CdSe/CdTe film stack. b) Higher magnification highlighting epitaxial growth and showing crystallographic defects passing through multiple layer boundaries.

A large number of multilayer absorbers covering a wide parameter space were deposited. A representative as-deposited CdSe/CdTe multilayer stack is shown in Figure 1. Several takeaways are apparent.

The intensity of the backscattered electron signal is determined by the atomic number. As heavier elements scatter incident electrons more strongly than lighter elements, CdTe appears brighter than CdSe. Individual CdSe and CdTe layers are clearly visible in the FE-SEM image. Boundaries between layers are very sharp, indicating that little intermixing of species occurs during deposition.

The first CdSe layer deposited on the TCO forms nanoscale grains, while the CdTe deposited on the initial CdSe layer has significantly larger grains. As this behavior follows previously reported similar observations [24–26] and this part of the layer stack is rather standard, the CdSe is assumed to grow in the hexagonal wurtzite phase while the CdTe is cubic zincblende. Interestingly, however, the subsequent ~100-nm CdSe layers deposited on CdTe grow completely epitaxially on the CdTe grains beneath, and are therefore assumed to grow in the cubic phase. These assumptions are corroborated by X-ray diffractograms of multilayer stacks prepared in a similar way to this sample, shown later in Figure 6a. All diffraction peaks can be assigned to the cubic phase of either CdTe or CdSe; no additional peaks are visible which could correspond to the hexagonal phase of either. In Figure 1, it is clear that many vertical grain boundaries pass through layer interfaces uninterrupted, and most individual large grains in the bulk have several layers of CdSe and CdTe within them. Even crystallographic defects such as twins are also seen to pass straight through layer interfaces in many places (Figure 1b).

Of course, the most striking feature is the significant number of voids, ranging from tens to several hundred nanometers in diameter. Film porosity is generally considered to be detrimental, and although significant recrystallization and grain growth would be expected to occur during CdCl_2_ annealing, it is no guarantee that this process would eliminate this porosity. Absorber voids in a finished cell would act as non-conductive recombination centers, reducing voltage and fill factor in particular [23,33,34]. Additionally, as-deposited porosity may lead to higher surface roughness post-CdCl_2_ than would otherwise occur, and even to pinholes in extreme cases. Prevention of porosity in the as-deposited films is therefore quite important.

**Figure 2.**
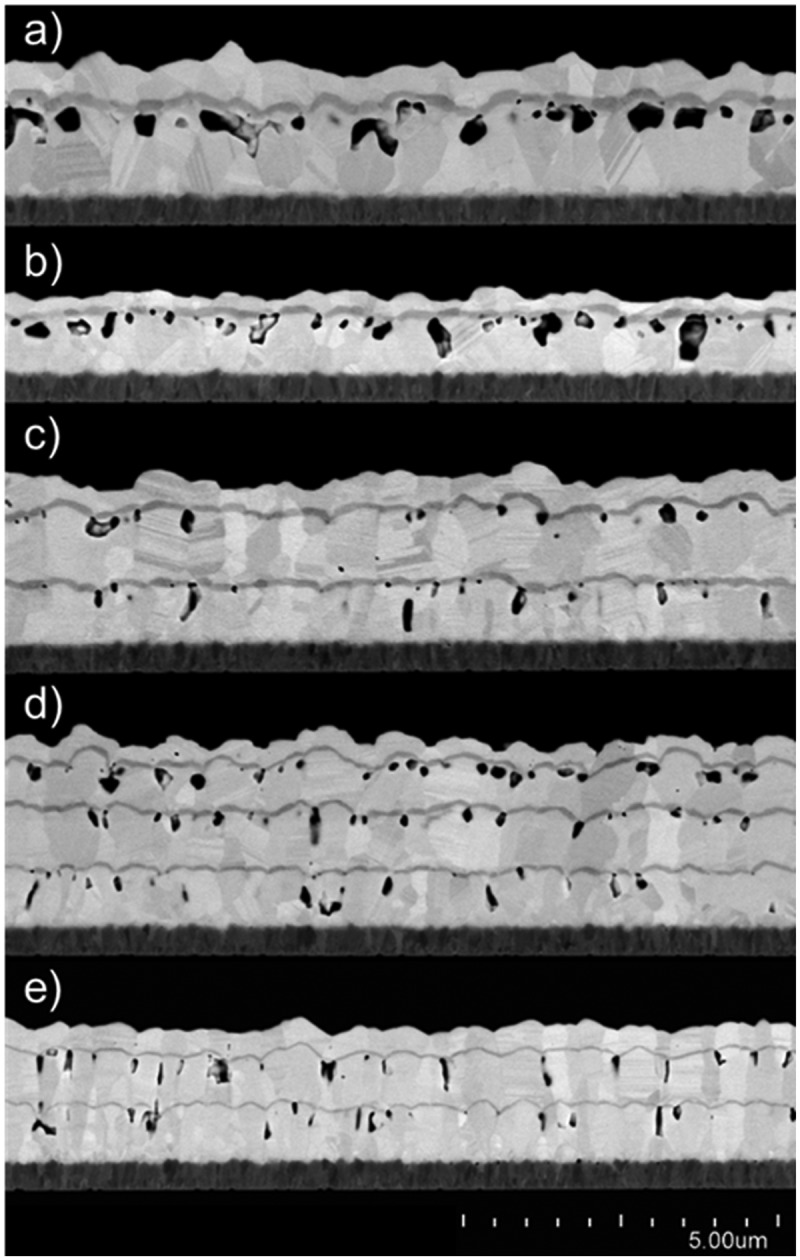
As-deposited multilayer CdSe/CdTe films under a range of processing conditions, all showing significant porosity. Starting from a) initial conditions, the films were deposited with b) reduced substrate temperature, c-d) increased substrate transport speeds, and e) an extended waiting time between each layer deposition. The number of layers and total thickness varies between samples.

Several variations in processing were attempted to eliminate or reduce this porosity; some of these are documented in cross-sectional FE-SEM images in Figure 2. From the initial process parameters (a), modifications included reduced substrate temperature (b), two progressively increasing substrate transport speeds (c,d), and an extended waiting time between each layer deposition (e), all in an attempt to reduce the temperature of the deposited film. The number of layers, overall thickness, and relative proportion of CdSe and CdTe vary somewhat between these samples. While these attempts yielded some differences in film structures such as grain size, as well as the apparent amount and geometry of voids, all failed to prevent significant void formation.

### Effect of CdCl_2_ anneal

**Figure 3.**
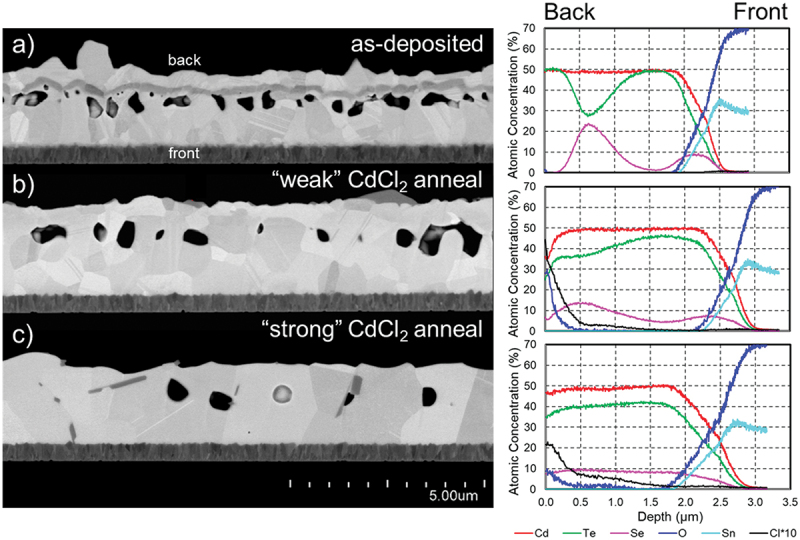
A representative porous multilayer film a) as-deposited, b) after a ‘weak’ CdCl_2_ anneal, and c) after a ‘strong’ anneal at higher temperature and longer time. Elemental concentration depth profiles are shown for each condition.

As the intention is to form a uniform CdSeTe layer through interdiffusion during CdCl_2_ annealing, the efficacy of this treatment must be examined. Three films with the same deposition configuration were investigated with FE-SEM cross-sections and GD-OES profiles, shown in Figure 3: the film as-deposited, after a ‘weak’ CdCl_2_ anneal, and after a ‘strong’ CdCl_2_ anneal. The ‘weak’ annealing condition involves a soaking temperature of <435°C for <20 min, while the ‘strong’ condition utilizes >435°C for >20 min with the amount of CdCl_2_ doubled to compensate, with not more than 20°C and/or 10 min difference between the two. In this material system, stronger CdCl_2_ annealing conditions generally facilitate grain reformation and intermixing of atomic species, which directly pertains to closing of pores and diffusion of selenium to form a uniform alloy. These two annealing conditions were therefore chosen to observe both an intermediate and final stage of film reformation. Both conditions are within the typical range of Cd(Se)Te device processing, though the ‘strong’ condition is rather aggressive for a film of this thickness. An example with a single intermediate CdSe layer is chosen so that diffusion behavior is easier to observe.

The as-deposited absorber looks typical, with a significant density of large pores present within the CdTe layer just below the CdSe interface. Grain size is rather small, with an average lateral width of 0.5 µm. The ~250-nm intermediate epitaxial CdSe layer is clearly visible, while the thinner hexagonal-phase layer at the front interface is more difficult to discern, and is comprised of very small grains.

Elemental distributions are shown in the GD-OES results. The back of the film stack is on the left and the front is on the right. The transparent conductive oxide (TCO) is clearly visible in the tin and oxygen signals. Note that the interface between the absorber and TCO is much more abrupt in reality than indicated by GD-OES. Surface roughness, the irregular shape of the sputter crater, and the dissimilar sputter rates of CdSeTe and SnO_2_ all contribute to the apparent stretching of the transition between these materials as seen by GD-OES. The two CdSe layers are clearly visible in the selenium profile. The tellurium signal of course inversely follows the selenium signal, and the cadmium signal is constant.

After a ‘weak’ CdCl_2_ anneal, the appearance of the film in cross-section changes significantly. The individual CdTe and CdSe layers are no longer discernible. The average lateral grain size has increased to 0.9 µm, but grains are still relatively small. Many large voids are still present; the voids seemed to have coalesced somewhat, as they are larger but fewer in number. The number of crystallographic defects has been reduced considerably. The concentrations of selenium and tellurium in the GD-OES profile have become more homogeneous, but the two selenium peaks are still clearly discernible. Chlorine and oxygen are now visible at the back; these are cadmium oxychloride residues which would typically be removed during full device processing. As with the TCO at the front, the oxychloride/absorber interface width is heavily exaggerated in GD-OES, although at this interface it is simply roughness of the back surface which smears the apparent boundary between the layers. Chlorine is also visible within the absorber. All in all, the incomplete mixing and incomplete recrystallization both convey that these annealing conditions are insufficient.

Film appearance changes even more after the ‘strong’ CdCl_2_ anneal. Grains are now much larger, with an average lateral size of 1.7 µm. Perhaps more significantly, most grains appear to be through-grains which span the absorber’s entire ~2.5 µm thickness. Voids are still present, though their concentration has been reduced further from the ‘weak’ anneal condition. There additionally now appear to be agglomerations of cadmium oxychloride residues within the absorber, which likely started as voids which then filled with CdCl_2_ and oxidized during annealing. GD-OES reveals that the concentrations of selenium and tellurium are now quite consistent throughout the absorber, with the CdSe fraction varying only between the extremes of ~17% at the front and ~20% at the back. Chlorine distribution is also significantly more uniform than the ‘weak’ anneal condition.

This investigation highlights two important findings about CdCl_2_ annealing on multilayer absorber stacks such as this. First, even in this fairly nonuniform as-deposited condition with only a single intermediate CdSe layer, the elemental distribution within the ~2.5 µm absorber becomes very uniform after a sufficiently strong CdCl_2_ treatment. Second, even with an anneal strong enough to substantially recrystallize the material, voids remain, both hollow and filled with CdCl_2_ residues. Porosity is expected to negatively impact device performance [23,33,34], so it is vital to dramatically reduce the prevalence of voids in the as-deposited state if the multilayer deposition approach is to be feasible.

### Porosity-free deposition conditions

**Figure 4.**
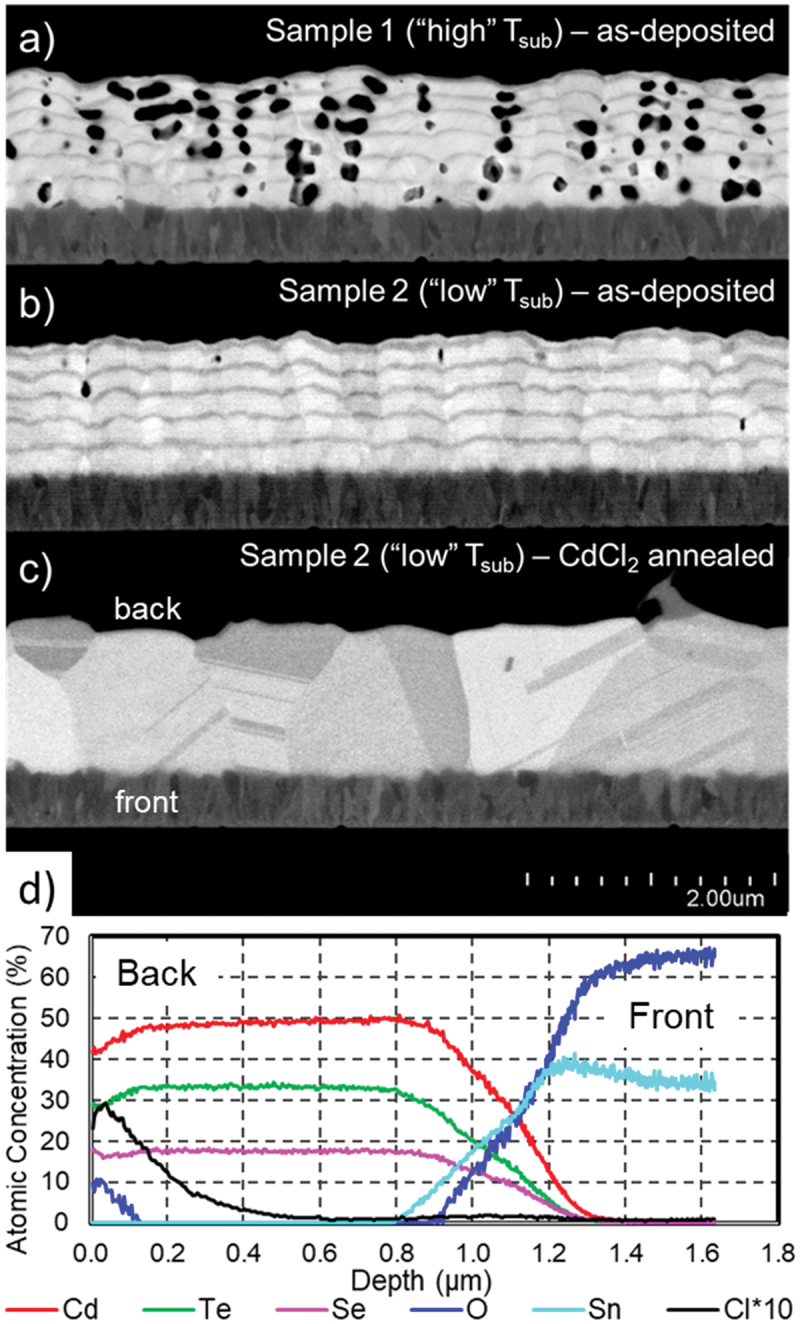
As-deposited exceptional multilayer absorbers deposited at a) ‘high’ and b) ‘low’ substrate temperatures, showing dramatic difference in porosity. c) ‘low’ substrate temperature film after the ‘strong’ CdCl_2_ anneal conditions as in Figure 3c, with d) corresponding elemental concentration depth profile.

By investigating well outside the usual processing space, conditions were eventually identified which form porosity-free multilayer films. Two elucidative film stacks are shown in Figures 4a,b, designated ‘sample 1’ and ‘sample 2’. Both films are deposited under fairly unorthodox conditions, with a fast substrate transport speed, significant waiting periods between each layer deposition, and unusually low source temperatures, particularly of CdSe. Both films are exceptionally stratified, with 6 and 5 intermediate CdSe layers, respectively. This is in part necessitated by the very low deposition rates under the utilized conditions, but also brings the benefit of high homogeneity in the as-deposited state.

The only meaningful difference in processing between the samples is 50°C lower substrate temperature setpoint used during deposition of sample 2. Nevertheless, the porosity of sample 1 is extreme, at 21.2% ± 1.1%, while sample 2 is fully dense, with a porosity 0.4% ± 0.1%. This large difference in film appearance demonstrates that void-free deposition unfortunately requires precise process adjustment and confirms that void formation is a consequence of high film temperature, which is elaborated on in the next section. As substrate heater setpoints are over 100°C lower than even the lowest source heater temperature, the actual temperature of the substrate is surely higher than the setpoint temperature during active deposition, and is influenced non-trivially by substrate *and* source heater temperatures, the amount of time spent under a source, and the length of time it has to cool off in between layer depositions. The temperature of the deposited film surface is no doubt even higher than the substrate. However, while the relationship is complicated, a lower substrate setpoint will nevertheless result in an overall lower substrate temperature during deposition, which appears to be the critical metric.

**Figure 5.**
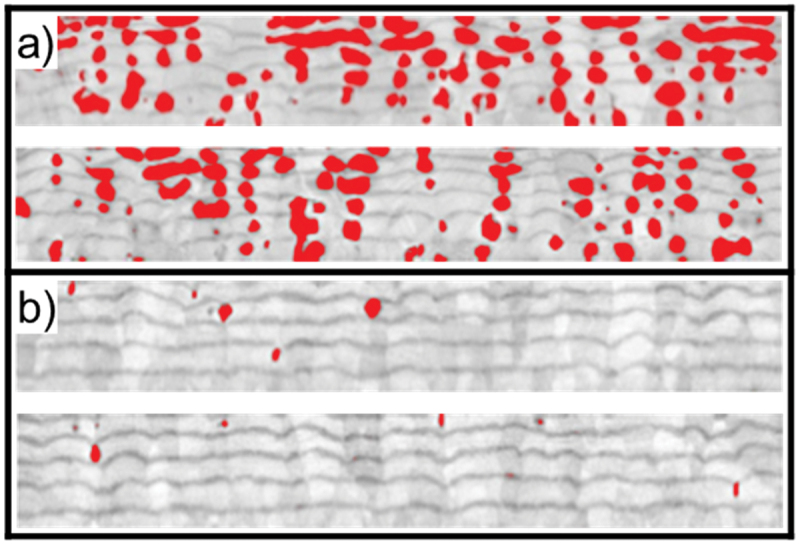
Voids identified by grayscale thresholding for porosity quantification of a) ‘sample 1’ and b) ‘sample 2’.

Porosity evaluation was done by quantitative image analysis using the ImageC software package. FE-SEM cross-section micrographs with porosity appearing as black were binarized with a threshold in grayscale and the ratio of the porous area in relation to the area of the whole absorber layer was calculated. This calculation was performed on two images from two positions on the same sample and averaged, as shown in Figure 5.

Sample 2 is a paragon of the multilayer approach. The as-deposited film shows many layers of remarkable uniformity. A few voids are still visible, but in such a small amount as to be considered negligible. Grains appear columnar, typically with many CdTe and CdSe layers within. Figure 4c shows this film after a CdCl_2_ anneal, during which dramatic restructuring occurs. The exact same annealing parameters as the ‘strong’ condition shown in Figure 3c were used. As in Figure 3c, the layers are no longer distinguishable and the grain size is significantly larger. The few voids in the as-deposited state have completely disappeared. Of particular note is the structure of the grains, which appears conducive to good cell performance. Most grains are similar in size, extend through the entire absorber, and are rather square: the average film thickness is 1.1 µm and average lateral grain size is 900 nm. There are almost no grain boundaries perpendicular to the carrier transport path. Thickness fluctuations are low, and the back surface is remarkably smooth. Elemental distribution from GD-OES is shown in Figure 4d; film composition is very homogeneous, constituting a uniform CdSe_x_Te_1-x_ film of x ≈ 0.35.

**Figure 6.**
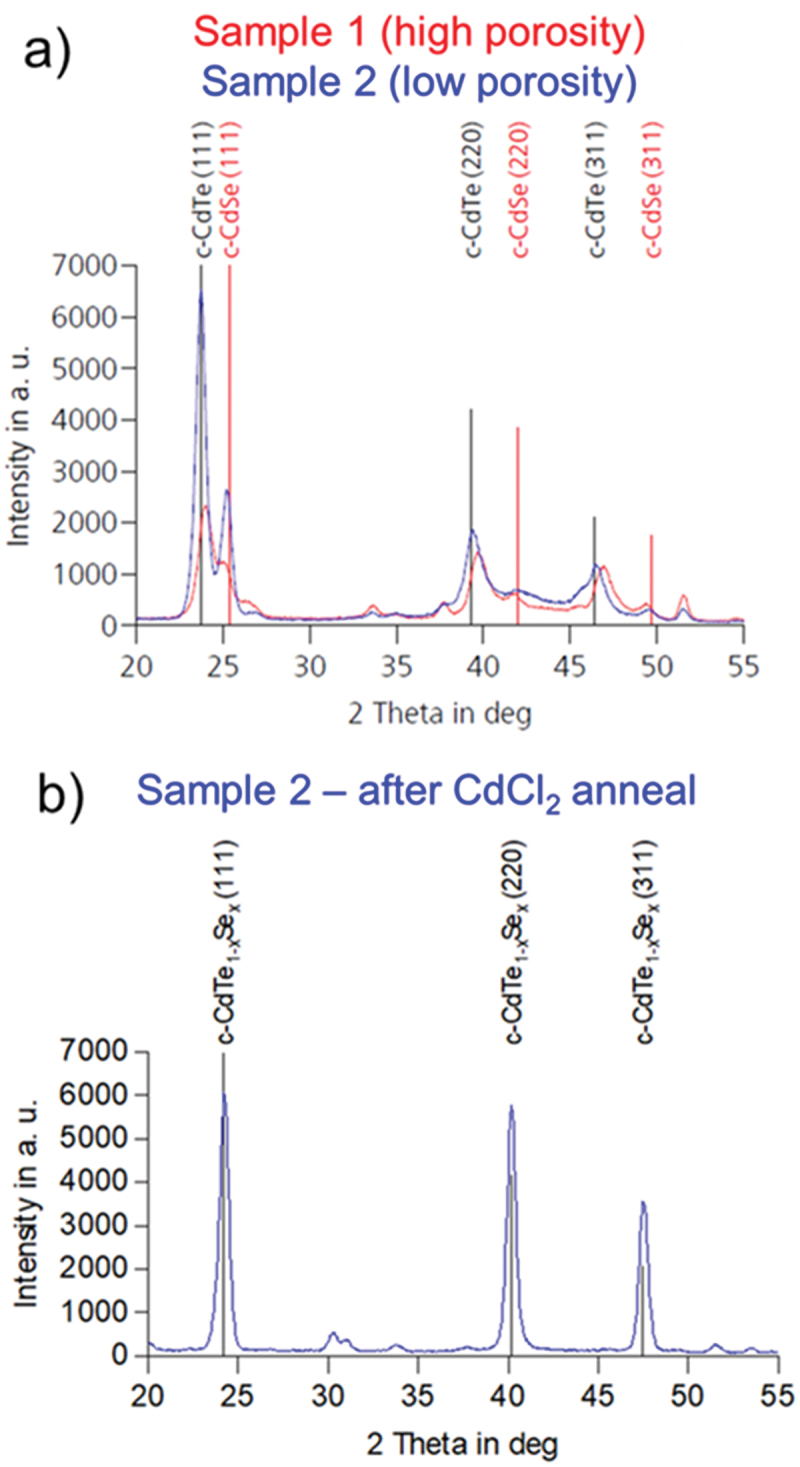
a) XRD diffractogram of as-deposited high and low porosity films shown in Figure 4. The high porosity film shows a slight shift in CdTe and CdSe peak positions, indicating interdiffusion. b) XRD diffractogram of CdCl_2_-annealed film, showing a single set of peaks corresponding to the cubic phase of the ternary Cd(Se,Te) alloy.

All three pictured films were investigated with glancing angle X-ray diffraction. The diffraction patterns of the as-deposited stacks are shown in Figure 6a; diffraction peak positions of pure cubic CdTe and cubic CdSe are also shown, taken from powder diffraction files from the ICDD database [35]. Most of the additional small peaks are attributable to SnO_2_. It is clear that in both samples there are exactly two sets of diffraction peaks corresponding to the cubic zincblende phases of CdTe and CdSe. These distinct species are also clearly visible as material contrast in the cross-sections of the as-deposited stacks.

After CdCl_2_ annealing, only a single set of peaks corresponding to the ternary Cd(Se,Te) phase is seen, shown in Figure 6b. This agrees with the FE-SEM micrograph, in which material contrast is no longer visible. As the Cd(Se,Te) material system obeys Vegard’s law [36], the lattice parameter *a* of the resulting alloy can be used to determine its composition. Lattice parameters can be calculated from diffraction peak positions according to the Bragg equation [37]. From the diffraction peaks in Figure 6b, a lattice parameter of 0.6332 nm was calculated, which corresponds to an atomic selenium content of 17.8%. This agrees very well with the ~35% CdSe fraction determined from GD-OES.

**Figure 7.**
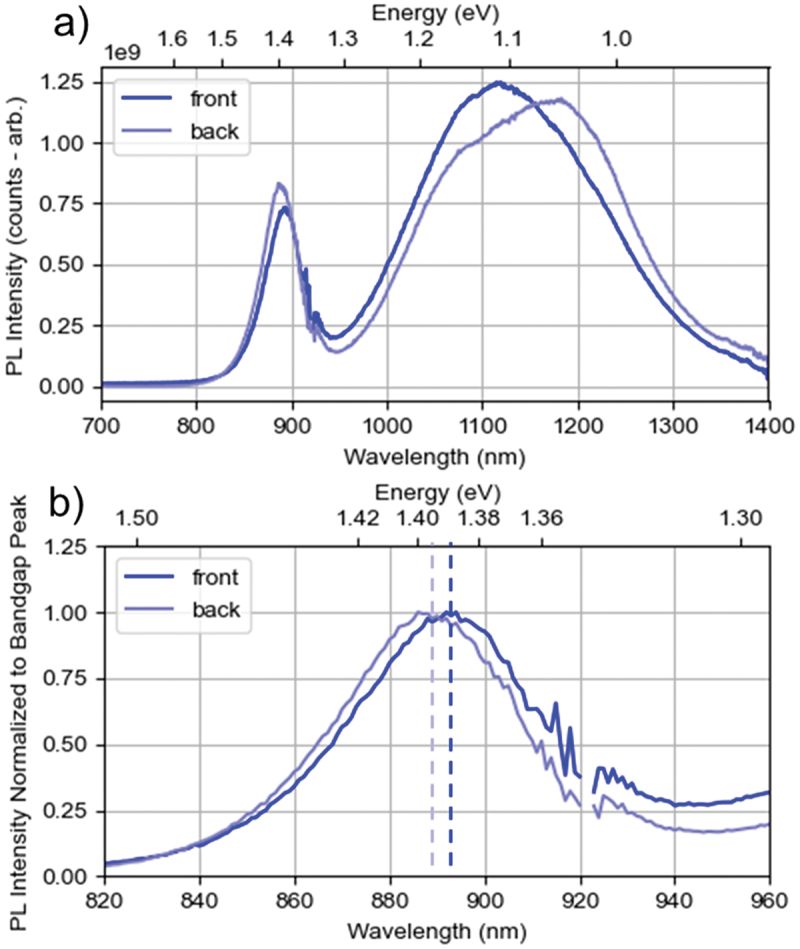
a) Photoluminescence emission spectra of the CdCl_2_-annealed film in Figure 4c measured from the front and back surfaces, and b) the same spectra focused on the band-to-band emission peak and normalized to better compare emission peak positions.

The photoluminescence emission spectra measured from the front and back sides of the annealed sample are shown in Figure 7. PL profiles are constructed from two spectra obtained using a UV/VIS detector (PicoQuant PMA Hybrid Si PMT, Germany) and an NIR detector (Hamamatsu H10330C-45 InP/InGaAsP PMT, Japan). Calibration to a reference light source enables both consistent scaling of intensity units between the two spectra and correction for uneven detection sensitivity over the detectors’ operational range. Both spectra can therefore be combined into a single spectrum covering the range 700 nm−1400 nm with the correct shape (the transition between the two is visible around 920 nm). Several points were measured on each side, and the most representative spectrum is shown. The band-to-band emission peak indicates a bandgap of ~1.39 eV from the front and ~1.395 eV from the back. This is essentially the lowest point of the bowed bandgap distribution of the CdSe_x_Te_1-x_ system [38], corresponding to x ≈ 0.35. The slight bandgap peak emission shift highlighted in the focused plot in Figure 7b indicates a small variation of the selenium distribution through the absorber thickness, but not more than x±0.05. The significant broad sub-band emission which eclipses the band-to-band peak is characteristic of high-selenium CdSeTe films. There is no doubt as to the composition and homogeneity of the annealed layer; GD-OES, XRD, and photoluminescence all indicate a uniform CdSe_x_Te_1-x_ alloy with x ≈ 0.35.

Although alloying with selenium near the front interface improves CdTe cell performance, replacing CdTe entirely with high-selenium CdSeTe such as in this film will produce poor-performing cells. High selenium content has been proven to cause implied voltage loss through subband defect states [14,39–41], to reduce carrier mobility [30,31], and to impede p-dopability with copper [38,42]. All of these effects can be quite significant, all compromise cell performance, and all intensify with higher selenium amount. CdSe_x_Te_1-x_ is also potentially n-type at sufficiently high x[14], which would necessitate a completely different cell design.

Ultimately, it should not be expected that good or even functional cells can be produced by simply swapping one absorber material for another in a structure heavily optimized for a certain material. CdSeTe has simultaneously many promising *and* inauspicious indicators, so more work will be needed either to identify device design and processing strategies which synergize with this material’s properties or to conclusively determine that this material is unsuitable as an absorber. Such work will require repeatable, configurable deposition of high-quality CdSeTe films, such as described in this manuscript. As no working cell fabrication process has yet been identified, PV device data is not included.

## Discussion

While it appears possible to make uniform CdSeTe films of excellent quality using the multilayer deposition approach, the tendency towards considerable void formation in as-deposited films is a significant impediment. Closer examination of these voids can help to identify their sources, and in the process suggest methods to suppress their formation.

There are several distinctive, recurring characteristics. The voids are nearly universally within the CdTe layers, at or very near the interface of a CdSe layer deposited afterward. Even very thin CdSe layers are typically completely intact and continuous, so voids do not form within CdSe. Although the voids may propagate downward with a significant depth into the CdTe layer, they nearly always have at least a tentative contact with the CdSe layer above. Voids are rarely present exclusively at the bottom of a CdTe layer. Likewise, the final (top) layer of CdTe almost never has voids.

Porosity in Cd(Se)Te has been observed and reported before. However, none of the explanations in the CdTe literature seem to adequately describe the phenomenon observed here. In this section, several hypotheses are evaluated, and most are dismissed.

### Miscellaneous phenomena

Film porosity has been reported in a few esoteric situations. Danielson et al. [11] describe void formation in CdTe when deposited on highly columnar CdSeTe layers. This is attributed simply to the difficulty of continuous deposition on such an unusually rough material. Colegrove et al. [20] describe void formation in CdSeTe due to chlorine migration during unconventional CdCl_2_ treatments. Hatton et al. [43] report large pores and blistering due to the escaping argon process gas implanted in sputtered CdTe films [44]. None of these interesting and unusual circumstances applies to the present observations.

### Kirkendall effect

One commonly identified mechanism of void formation in Cd(Se)Te films is through the Kirkendall effect [34,45,46]. At suitably high temperatures for diffusion, different atomic species in contact with one another diffuse across their common interface at different rates [47,48]. This can cause an accumulation of the faster-diffusing species into one region of the material, while the vacancies which this species fills diffuse in the other direction. A sufficient concentration of these vacancies can form agglomerations which eventually appear as larger-scale structures observed as voids.

In this particular alloy system, selenium diffuses faster into CdTe than tellurium diffuses into CdSe [34]. This suggests that Kirkendall void formation should occur within CdSe. As porosity in the multilayer films is observed entirely within the CdTe and the CdSe layers always appear intact despite their low thickness, it can be concluded that Kirkendall voiding is not responsible for the observed behavior. Additionally, Kirkendall-effect-attributed voiding in this material is generally seen within CdSeTe formed by diffusion *after* CdCl_2_ treatments, while the multilayer film voids observed here appear in un-diffused CdTe *before* any high-temperature CdCl_2_ annealing.

### Lattice shrinkage

Although the as-deposited CdTe and CdSe layers are clearly distinguishable in the cross-section images with abrupt interfaces, it is possible that some amount of interdiffusion is occurring between the layers and forming CdSeTe from the heat of the deposition itself [12]. As the lattice size of cubic CdSe_x_Te_1-x_ decreases as x increases, interdiffusion would cause the CdTe to shrink after it has been deposited; the difference in unit cell volume could feasibly manifest as porosity. In this situation, voids would form at the interface of the two binary materials where formation of the ternary alloy would occur. It is even conceivable that the formation of CdSeTe would occur predominantly or exclusively within CdTe. Void formation would therefore be within CdTe at the interface with CdSe, as is observed.

To test this hypothesis, the X-ray diffractograms from Figure 5a can be analyzed further; these films are illustrative as they show a dramatic difference in porosity but are otherwise very similar. In sample 2 (no porosity), the visible peaks correspond very closely to those of CdTe and CdSe, indicating distinct CdTe and CdSe layers with no significant intermixing. In sample 1 (high porosity), all CdTe peaks shift distinctly to higher 2θ values, while CdSe peaks shift in the opposite direction. This implies a degree of CdTe/CdSe interdiffusion. Both samples were measured with X-ray fluorescence spectroscopy (described in section 4.4) to determine elemental composition, and both have nearly identical selenium concentrations: 16.4% ± 1.4% in sample 1 and 16.5% ± 1.1% in sample 2. The shift in diffraction patterns between the samples can therefore not be explained by the overall composition.

The degree of intermixing can be quantified from the lattice parameter a, once again calculated from diffraction peak positions according to the Bragg equation [37]. The effect of film stress on diffraction peak shifts is neglected. Calculated from the mean of the (111), (220), and (331) CdTe peaks, the lattice parameter of sample 2 (no porosity) is 0.6476 nm, very close to the expected value of pure CdTe. Correspondingly, the lattice parameter of sample 1 (high porosity) is slightly lower at 0.6421 nm. The unit cell volume of sample 1 is therefore approximately 2.5% smaller than sample 2. While possibly a contributing factor, this is not nearly enough to account for the ~21% mean porosity observed in sample 1. Lattice shrinkage due to intermixing can therefore not explain the observed void formation on its own. This does not rule out the possibility of an important indirect contribution, such as localized volume reduction serving as nucleation sites from which resublimation of larger pores is initiated, as discussed below.

### Re-sublimation

**Figure 8.**
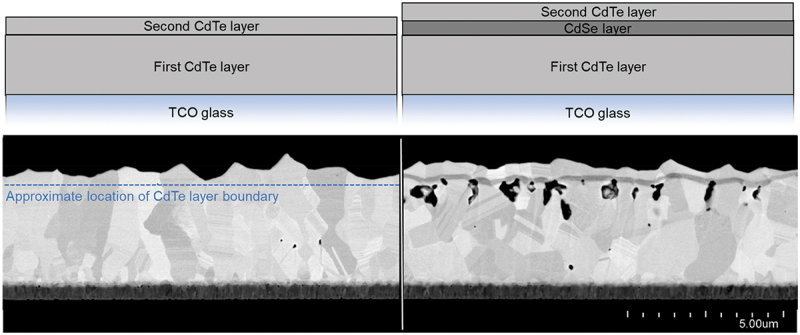
Schematics and FE-SEM images of layer stacks used for XRF investigation. One stack has an intermediate CdSe layer while the other does not; the total geometric thickness of CdTe is equivalent, but the sample with CdSe has large voids, indicating loss of CdTe.

The above hypotheses all generally describe a fixed amount of material which is rearranging itself. Another possibility is that voids signify that material has left the layer stack entirely. To investigate this possibility, two film stacks were prepared, both with a single oscillation away from the CdTe source and back. In one sample, the substrate oscillated to the CdSe source, and in the other sample, the substrate oscillated into an empty region of the chamber at idle temperature. All other process conditions between the samples were identical, including substrate/source temperatures, transport speed, chamber pressure, and the number of oscillations. A schematic of the simple stacks as well as FE-SEM cross-sections are shown in Figure 8. Material interfaces are clearly visible in the CdTe/CdSe/CdTe stack. In the CdTe/CdTe stack, the second CdTe layer grows epitaxially on the first, so there is no visible boundary between the two; its approximate location is indicated by the dashed line. As both stacks are exposed to the CdTe source at the same temperature for the same time in total, both films would be expected to have the same amount of CdTe overall, in the absence of other effects. Indeed, the geometric CdTe layer thicknesses determined from the cross-sections are essentially the same: 3.6 µm ±0.1 µm and 3.56 µm ±0.13 µm for the stacks without and with the CdSe layer, respectively. However, the significant porosity in the CdTe/CdSe/CdTe stack would therefore suggest that the amount of CdTe in this film is less than in the CdTe/CdTe stack, which has negligible porosity.

To quantify this difference, both samples were measured with X-ray fluorescence (XRF) spectroscopy, in which core electrons are ejected through exposure to high energy X-rays. Valence electrons release energy in the form of X-rays to fill these vacancies, which is detected. From the resulting fluorescence spectrum, elemental composition and concentration can be determined by deconvolution with each element’s characteristic radiation signature. Using XRF, the number of tellurium atoms in the sample without the CdSe layer was determined to be approximately (5.20 ± 0.15)×10^18^ cm^−2^. In the sample with the intermediate CdSe layer, this quantity was only (4.48 ± 0.14)×10^18^ cm^−2^. This can only mean that the total amount of CdTe in the second film is less than the first. As the same amount of CdTe *should* have been deposited in both cases, the simplest conclusion is therefore that high temperatures during deposition of the intermediate CdSe layer cause resublimation of the already-formed CdTe beneath. The missing CdTe material manifests as porosity.

This experiment demonstrates conclusively that it is the deposition of the CdSe layer which is the source of the porosity in CdTe. CdTe resublimating from the high CdSe source temperature would form voids just beneath each CdSe layer, which is exactly what is observed. While the identity of the escaped species is clear, the precise mechanism and timing of the resublimation is still uncertain. In the cross-sections depicted in this manuscript, while most CdSe layers appear intact, gaps in CdSe are visible in a few places through which CdTe could potentially have escaped. In other places, the CdSe is continuous but thinner above the void, as if the CdSe deposition was locally disrupted by CdTe vapor escaping. In still other places, the pores appear to have no influence on the CdSe layer above. Occasionally voids even appear far from the CdTe/CdSe interfaces. It is possible that, as each image is a thin slice, a large proportion of pores may have three-dimensional escape paths which are only visible in a different plane. Additionally, if void formation occurs concurrently with CdSe deposition, not all pores would necessarily require an escape channel, as the CdSe film can close after some amount of CdTe has sublimated, halting the process. Perhaps as the CdSe forms this ‘dome’ over the pore, it is supported by the vapor pressure of escaping CdTe.

If all species are deposited simultaneously at the same temperature, void formation through this resublimation mechanism should not occur, as films will simply form with an average growth rate and composition determined by deposition conditions. Indeed, sublimating the CdSeTe source material generally does not appear to produce porous films [11]. To avoid both porosity and the difficulty of working with premixed materials, co-sublimation of CdSe and CdTe from separate compartments within the same source crucible at the same temperature may be another method for deposition of CdSeTe films with flexible, tailorable stoichiometry.

## Conclusion

It has been demonstrated that high-quality uniform CdSeTe layers for CdTe photovoltaics can be produced by close-space sublimation of alternating CdSe and CdTe layers followed by a CdCl_2_ annealing treatment. Constituent atomic species readily diffuse to a homogeneous equilibrium with the help of CdCl_2_, and precise control over alloy composition is afforded. Such highly configurable films are useful for investigating the suitability as an absorber of CdSeTe, whose properties are strongly influenced by the relative fraction of CdSe.

However, there is a significant drawback. It has been revealed that this method has a strong tendency to form numerous voids in the as-deposited CdTe layers. Porosity ultimately arises from the higher sublimation temperature of CdSe compared to CdTe. It appears that deposited CdTe layers resublimate upon exposure to the higher-temperature CdSe source, leaving behind voids. It is only through careful adjustment of processing conditions that this effect can be suppressed, particularly through reduction of the CdSe source temperature. While recrystallization during CdCl_2_ annealing can remedy mild as-deposited porosity to some degree, it has been shown that severe porosity cannot be fixed by even a strong CdCl_2_ treatment. Although deposition conditions necessary to achieve pore-free uniform CdSeTe absorbers with this technique may be impractical to implement at the production scale, precisely configurable films are useful for fundamental material investigations, where this limitation is far less relevant.
